# Survey of Parental Use of Antimicrobial Drugs for Common Childhood Infections, China

**DOI:** 10.3201/eid2607.190631

**Published:** 2020-07

**Authors:** Leesa Lin, Stephan Harbarth, Xiaomin Wang, Xudong Zhou

**Affiliations:** London School of Hygiene and Tropical Medicine, London, UK (L. Lin);; University of Geneva Hospitals and Faculty of Medicine, Geneva, Switzerland (S. Harbarth);; Zhejiang University, Hangzhou, China (X. Wang, X. Zhou)

**Keywords:** antimicrobial resistance, bacteria, self-medication, antibacterial agents, practice patterns, physicians, child, China, surveys and questionnaires, pediatrics, common cold, parental

## Abstract

In a large-scale survey of 9,526 parents in China, we investigated antimicrobial drug use for common childhood infections. Of children with self-limiting conditions, formal care was sought for 69.2%; of those, 53.4% received drug prescriptions, including 11.2% from parental demands. Where drugs were taken without prescriptions, 70% were from community pharmacies.

China accounts for half of global consumption of antimicrobial drugs (*1*,*2*) and reports high rates of antimicrobial resistance, especially among children (*3*,*4*). Despite current regulations requiring a prescription to obtain antibiotics, unsupervised use in China is pervasive (*3*,*4*). To date, the few studies available on inappropriate administration to children beyond clinical settings have been limited in scope to small-scale data in one geographic area (*3*,*5*–*7*). To inform control strategies, we investigated the prevalence of inappropriate antibiotic use for common childhood infections by parents across different geographic areas and economic development stages, overall parental knowledge about antimicrobial drug use and resistance, the impact of parental pressure for antibiotics on prescribing behaviors, and parental contribution to the overall use of antibiotics in children.

## The Study

Study methods, including sampling strategy and data collection, were previously reported (*8*). Our data came from a cross-sectional survey conducted during June 2017–April 2018, which recruited 9,526 parents with children 0–13 years of age across 3 provinces (Zhejiang, Shaanxi, and Guangxi) representing different geographic areas and economic development stages in China (*9*). We conducted multistage stratified random cluster sampling in provinces, prefecture-level cities, urban and rural areas, and local sampling sites that included primary schools (children 6–13 years of age), kindergartens (3–5 years), and community-based health centers (0–2 years). The Institutional Review Board at the Zhejiang University School of Medicine (approval no. ZGL201706-2) and London School of Hygiene and Tropical Medicine (approval no. 14678) reviewed and exempted the study protocol and survey.

**Figure 1:**
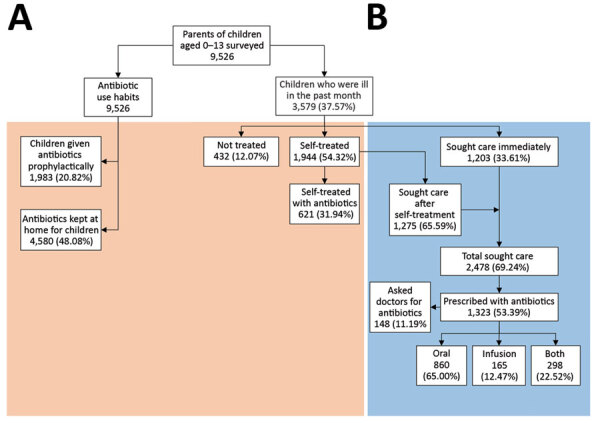
Use of antimicrobial drugs (antibiotics) for children by their parents (N = 9,526), China. Section A (left of dashed line) represents chronic antibiotic use in the previous year; section B (right of dashed line) indicates antibiotic use for common childhood illnesses in the previous month. Orange shading indicates treatment within community or household, outside of a clinical setting. Blue shading indicates use within a clinical setting.

We recruited parents through their children, and all participants gave informed consent. We distributed a questionnaire to parents who self-identified as the primary caregiver and healthcare decision maker for the children; parents completed the questionnaire electronically. We collected data on parental sociodemographics, knowledge about antibiotic use and resistance, and last episode of illnesses and treatments experienced by the child within a month (Figure 1). 

We calculated an 89% response rate by dividing the number of parents who completed the survey by the number of parents who were invited to take the survey (13,680/15,424 ). For data quality control, we used measures (including trap questions and IP address control) to detect random responses or duplications, resulting in 70% (9,526/13,680) of the completed surveys verified as valid. We classified missing data (<11%) as missing-completely-at-random and excluded from the final analysis.

**Figure 2:**
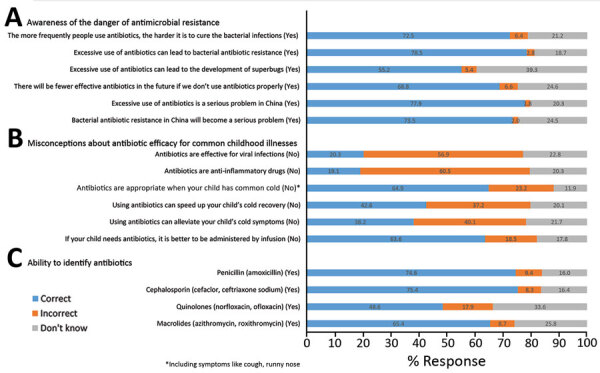
Answers to questions about antibiotics-related knowledge among parents in 3 representative provinces in China (N = 9,526). A) Knowledge of risks for antimicrobial resistance; B) understanding of drug efficacy for common illnesses; C) drug recognition. Correct answers are shown in parentheses.

We conducted descriptive analysis on the data, followed by unadjusted univariable analysis to examine the association between antibiotic prescriptions and parental demand and employed multivariable logistic regression and likelihood ratio tests for adjusted analyses, controlling for relevant sociodemographic variables. We observed profound regional differences in parental socioeconomic composition, antibiotic-use practices, and medical facilities used when children (age 5.8 years, SD + 3.6 years) were ill (Table). Most parents (55.2%–78.5%) were aware of the danger that overuse of antibiotics poses, yet confused antibiotics with inflammatory drugs or drugs effective for treating colds or alleviating symptoms (Figure 2). Among the respondents, 37.6% (3,579/9,526) self-reported that their children experienced a minor illness within the previous month, with some overlap between symptoms; 82.1% (2,938/3,579) reported that it was a common cold with rhinitis, nasal congestion, or cough; 47.7% (1,707/3,579) sore throat; 31.0% (1,108/3,579) fever; 12.5% (446/3,579) diarrhea; and 3.3% (119/3,579) otitis media. Among parents who reported to have self-treated sick children, 31.9% (621/1,944) reported medicating them with antibiotics from either a local pharmacy (57.0%, 354/621) or personal stock (33.3%, 207/621). Almost all household stock of antibiotics was reported to have come from leftover prescriptions (63.1%, 2,891/4,580) or over-the-counter purchases (35.3%, 1,619/4,580); community pharmacies accounted for 70% of antibiotics for self-medication of children.

**Table:** Characteristics of antibiotic prescriptions for children with common childhood illnesses in the previous month, China*

Study question	Children brought to medical facility, N = 2,478, no. (%)					
			Children prescribed antibiotics, n = 1,323 [53.39%]			
			No. (%)	OR	aOR (95% CI)†	p value‡
What was the medical facility used when seeking care for your child? (urban/rural)						**0.04**
Tertiary hospital	367 (14.8)		174 (47.41)	Referent	Referent	
Secondary/county hospital	1,057 (42.7)		592 (56.01)	1.41 (1.11–1.79)	1.43 (1.11–1.84)	
Community health centers/township hospital	719 (29.0)		373 (51.88)	1.20 (0.93–1.54)	1.17 (0.90–1.53)	
Private clinics/ village clinics	335 (13.5)		184 (54.93)	1.35 (1.00–1.82)	1.21 (0.89–1.65)	
When seeking care at the medical facility, did you ask the doctor for antibiotics?						**<0.0001**
No	2,292 (92.5)		1,175 (88.81)	Referent	Referent	
Yes	186 (7.5)		148 (11.19)	3.70 (2.57–5.34)	3.71 (2.56–5.38)	

*Bold text indicates a statistically significant difference (p<0.05). aOR, adjusted odds ratio; OR, odds ratio.  †Adjusted for children’s sex and age, urban/rural residence, and province. ‡Determined by likelihood ratio test.

Of 3,579 parents whose children were ill in the previous month, 69.2% (2,478/3,579) sought care for the child. Before seeing a doctor, 16.5% (410/2,478) of children had already been medicated with antibiotics at home; moreover, among them, 15.4% (63/410) of parents admitted to having then asked for more antibiotics at the facility. Among those for whom care was sought after parents medicated with antibiotics, 83.9% (344/410) were prescribed antibiotics, 17.2% (59/344) of them because of parental demand. Data showed the success rate of obtaining antibiotic prescriptions for children was 79.6% (148/186); parental demand for antibiotics was more likely to occur in lower-level hospitals than tertiary hospitals and was associated with ≈4-fold increase in prescribed antibiotics (Appendix Table).

Overall, 53.4% (1,323/2,478) of children for whom care was sought were prescribed antibiotics. The most commonly prescribed antibiotic classes were penicillins, macrolides, and cephalosporins, either alone or in combination. Differences emerged in prescription rates by type of healthcare facility, ranging from 47.4% (174/367) in tertiary hospitals to 56.0% (592/1,057) in county hospitals. More than 33% of children were administered intravenous antibiotics; about half of those infusions were combined with oral antibiotics.

Our data showed that, among the 3,579 children who had common minor childhood illnesses (mostly self-limiting) in the previous month, 621 were administered nonprescription antibiotics by their parents and 1,323 obtained a prescription, with 148 of those deemed inappropriate due to parental demand. We estimated that demand from parents contributed to 40% of antibiotic use on children for self-limiting illnesses. Although some doctors’ prescriptions (supply-side) might be considered appropriate, all antibiotic demands and nonprescription uses from parents were inappropriate.

## Conclusions

Overuse of medical care for self-limiting illnesses combined with a high prescription rate and a large population size drive high antibiotic consumption in China. We found that, of children for whom care was sought, 53.4% (1,323/2,478) received prescriptions for antibiotics; this proportion is at least twice as high as the official cap of outpatient prescriptions enforced in 2012 (*10*,*11*). Unsupervised administration of antibiotics in children in the household, 3–10 times higher in China than in some countries in Europe (*12*), is a serious problem that has persisted for 15 years (*13*). Despite a 2004 ban on nonprescription sales of antimicrobial drugs (*10*,*11*), participants in all sampled sites have been able to obtain them over the counter. 

Enforcing existing stewardship policies is an important step to reduce inappropriate antibiotic use in the community (*11*), as is a multifaceted program that addresses drivers of inappropriate use from both sides of healthcare system. Such a program should provide parents with education about antibiotic efficacy and care for childhood illnesses (*14*), corresponding to children’s developmental stages: prenatal care, vaccination, and kindergarten and primary school–age concerns. The program should also support healthcare providers by removing financial incentives to overprescribe medications and outpatient pediatric infusion services, enhancing clinical diagnostic capacity, and providing training on rational prescribing (*14*,*15*). Finally, interventions to improve effective patient–physician interaction and communication should consider both sides of the healthcare system (*14*).

This study is limited by its cross-sectional design; it cannot be used to establish causal conclusions and is subject to recall bias. We limited questions about healthcare-seeking behaviors to the month before the survey and recruited a large sample to reduce the risk for bias. Because we estimated antibiotic consumption by a snapshot survey and not by prescriptions or use, the true magnitude of misuse in children may be underestimated. Common childhood illness cases reported in this study were diagnosed by parents; such diagnoses may or may not reflect true prevalence of the diseases, yet were a key determinant of parents’ childcare behaviors that dictated healthcare decisions parents made for their children.

Appendix Additional information about parental use of antimicrobial drugs for common childhood illnesses, China.
