# Supplementary material for: Survey of Parental Use of Antimicrobial Drugs for Common Childhood Infections, China
Source: Emerg Infect Dis. 2020 Jul;26(7):1517–20. doi: 10.3201/eid2607.190631 (PMC7323518; doi:10.3201/eid2607.190631)
Supplement: Appendix — Additional information about parental use of antimicrobial drugs for common childhood illnesses, China. [file 19-0631-Techapp-s1.pdf]

Article DOI: https://doi.org/10.3201/eid2607.190631

Survey of Parental Use of Antimicrobial Drugs for Common Childhood Infections, China

Appendix

Appendix Table. Characteristics of study population, by geographic area, in study of parental use of antibiotics for childhood illnesses*

Characteristic	Total (%)	Province, region, national GDP rank† Zhejiang East, 5th	Shanxi Central-Northwest, 12th	Guangxi Southwest, 26th	Urban	Rural
Sampled size by geographic area	9,526 (100%)	2,924 (30.7)	3,355 (35.2)	3,247 (34.1)	5,265 (55.3)	4,261 (44.7)
What is the sex of your child?						
Male	4,943 (51.9)	1,511 (51.7)	1,683 (50.2)	1,749 (53.9)	2,729 (51.8)	2,214 (52.0)
Female	4,583 (48.1)	1,413 (48.3)	1,672 (49.8)	1,498 (46.1)	2,536 (48.2)	2,047 (48.0)
What is the age of your child? [mean, y (SD)]	5.84 (3.6)	6.19 (3.6)	5.62 (3.5)	5.74 (3.6)	5.77 (3.6)	5.92 (3.6)
What is your average household income? (RMB/mo)						
≤3,000	2,102 (22.1)	123 (4.2)	753 (22.4)	1,226 (37.8)	607 (11.5)	1,495 (35.1)
3,001–5,000	2,889 (30.3)	519 (17.8)	1,294 (38.6)	1,076 (33.1)	1,486 (28.2)	1,403 (32.9)
5,001–10,000	2,749 (28.9)	1,033 (35.3)	1,040 (31.0)	676 (20.8)	1,768 (33.6)	981 (23.0)
>10,000	1,786 (18.8)	1,249 (42.7)	268 (8.0)	269 (8.3)	1,404 (26.7)	382 (9.0)
What is your education level?						
Primary school or below	435 (4.6)	56 (1.9)	110 (3.3)	269 (8.3)	144 (2.7)	291 (6.8)
Middle school	2,763 (29.0)	515 (17.6)	1,053 (31.4)	1,195 (36.8)	990 (18.8)	1,773 (41.6)
High school	2,668 (28.0)	605 (20.7)	1,130 (33.7)	933 (28.7)	1,416 (26.9)	1,252 (29.4)
College or above	3,660 (38.4)	1,748 (59.8)	1,062 (31.7)	850 (26.2)	2,715 (51.6)	945 (22.2)
Have you received any medical education/training? (Yes)	1,122 (11.8)	345 (11.8)	436 (13.0)	341 (10.5)	736 (14.0)	386 (9.1)
During the past year, did you ask your children to take antibiotics prophylactically (e.g., when your children’s classmates were having a cold or flu)? (Yes)	1,983 (20.8)	489 (16.7)	924 (27.5)	570 (17.6)	1,062 (20.2)	921 (21.6)
Do you keep antibiotics at home for your children? (Yes)	4,580 (48.1)	1,333 (45.6)	1,950 (58.1)	1,297 (39.9)	2,668 (50.7)	1,912 (44.9)
Has your child been ill in the past month? (Yes)	3,579 (37.6)	1,021 (34.9)	1,294 (38.6)	1,264 (38.9)	2,035 (38.7)	1,544 (36.2)
During self-treatment at home, did you use antibiotics on your child? (Yes)	621 (31.9)	105 (18.8)	297 (42.7)	219 (31.8)	350 (30.6)	271 (33.8)
When seeking care at a medical facility, did the doctor prescribe antibiotics for your child? (Yes)	1,323 (53.4)	351 (49.0)	509 (56.6)	463 (53.7)	782 (56.5)	541 (49.4)
When seeking care at a medical facility, did you ask the doctor for antibiotics? (Yes)	186 (7.5)	56 (7.8)	61 (6.8)	69 (8.0)	98 (7.1)	88 (8.0)

*Sample size is 9,526 parents with young children. RMB, Renminbi.
†Source: National Bureau of Statistics China. http://data.stats.gov.cn
